# Treatment of cardiogenic shock with left ventricular assist device combined with cardiac resynchronization therapy: A case report

**DOI:** 10.1186/1749-8090-5-54

**Published:** 2010-07-02

**Authors:** Håvard Keilegavlen, Jan Erik Nordrehaug, Svein Faerestrand, Rune Fanebust, Reidar Pettersen, Rune Haaverstad, Vegard Tuseth

**Affiliations:** 1 Department of Heart Disease, Haukeland University Hospital, Bergen, Norway

## Abstract

Cardiogenic shock has a poor prognosis with established treatment strategies. We report a 62 years old man with heart failure exacerbating into refractory cardiogenic shock successfully treated with the combination of a percutaneous left ventricular assist device (LVAD) and subacute cardiac resynchronization therapy (CRT) implantable cardioverter-defibrillator device (CRT-D).

## Background

The mortality rate in patients with cardiogenic shock is still very high [1]. Medical therapy has symptomatic effects, but has no proven reduction of mortality. Percutaneously placed LVAD is an option for selected groups of these patients. The percutaneous microaxial blood pump, Impella LP 2.5^® ^(Abiomed; Aachen, Germany) can be rapidly deployed with low complication rates and have improved hemodynamic effects compared with the intraaortic balloon pump (IABP) [2-4]. Furthermore, in selected patients with stable heart failure, CRT is proven to relive symptoms and improve outcomes [5]. The potential efficiency of acute and subacute CRT treatment in patients with cardiogenic shock has to our knowledge not been studied.

## Case presentation

A previously healthy 62 years old man who had experienced reduced exercise capacity for the last 6 months was admitted to the local hospital after 2 weeks of increasing dyspnoea. Echocardiography revealed biventricular dilatation, reduced wall thickness, asynchronous left ventricular (LV) contraction and left ventricular ejection fraction (LVEF) of 10%. ECG showed left bundle branch block (QRS width 170 msec). The clinical condition deteriorated rapidly into a cardiogenic shock. Multiorgan failure developed including hepatic dysfunction and renal impairment. The following day, he was transferred to our hospital for LVAD therapy. An Impella LP 2.5^® ^was percutaneously deployed, and the mean arterial pressure immediately improved from 50 mmHg to 70 mmHg and the vasopressor drugs could be stopped. Coronary angiography showed normal coronary arteries. The patient clinically improved and INR and s-creatinine normalized during the first three days.

**Figure 1:**
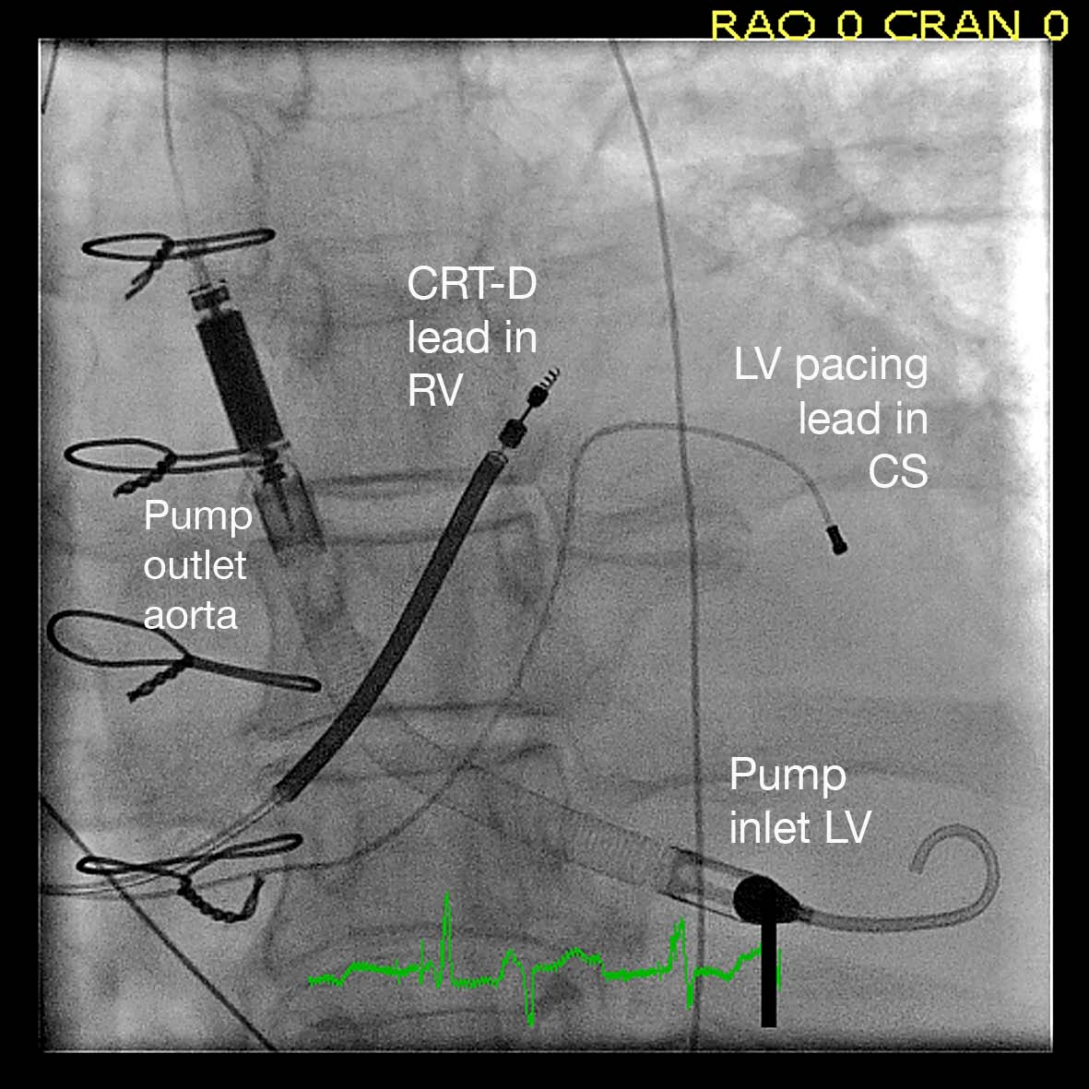
**Implanted Impella Recover^® ^LP 5.0**. The right ventricle (RV) pacemaker/defibrillator lead is located posteriorly in the septal part of RV outflow tract. The left ventricular (LV) pacing lead placed epicardially in a mediolateral branch from the coronary sinus (CS). The atrial lead is not seen in the image.

After five days LVEF was still only 10% and blood pressure could not be sustained without LVAD support. Due to refractory decompensated heart failure and severe asynchronous LV contraction with left bundle branch block, a CRT-D (Medtronic Insync Sentry 7298) was implanted on vital indication. The procedure was complicated by pericardial tamponade not responding to pericardiocentesis. Sternotomy was required to repair a perforation of the right atrium with direct suture. In order to permit prolonged LAVD support and increase pump delivery, Impella LP2.5^® ^was on day 6 after admission replaced through a surgical incision with an Impella LP 5.0^® ^with a maximum flow rate of 5.0/min (Figure 1). Ventilator treatment and LVAD support were continued for a total of 22 days. Transient infections were treated with antibiotics. There were no signs of renal impairment, central neurological deficits or mental impairment. The CRT-D was optimized by adjustments of the atrioventricular delay and interventricular timing of pacing guided by echocardiography. At outpatient control after four months the patient was in New York Heart Association (NYHA) functional class IIb with LVEF of 22% and maximal oxygen uptake during exercise was 13.9 ml/kg/min.

## Discussion

In the reported case, the patient presented with untreated severe decompensated dilated cardiomyopathy with hemodynamic instability exacerbating into cardiogenic shock refractory to standard intensive medical treatment. IABP has been the most widely used mechanical hemodynamic assist device. In spite of beneficial hemodynamic effects and a low complication rate, no randomized clinical studies have shown reduction of mortality [2]. Other available hemodynamic support strategies include surgical cardiopulmonary support (CPS) and different percutaneous LVAD systems (i.e. the TandemHeart^® ^and the Impella LP 2.5/5.0^®^). The Impella LP 2.5^® ^is inserted via the femoral artery and advanced retrogradly into the left ventricle. An electromagnetic motor draws blood from the inflow port in the left ventricle to the outflow port in the proximal ascending aorta close to the inlet of the coronary arteries. Small studies comparing IABP and Impella in cardiogenic shock may indicate beneficial hemodynamic effects of the percutaneous LVAD [3,4]. Experimental studies have shown that Impella LP 2.5^® ^may sustain vital organ perfusion even during cardiac arrest [6]. Thus, the percutaneous LVAD may have potential to significantly improve hemodynamics in selected critically ill patients.

CRT improves symptoms and reduces mortality by 36% in patients with ischemic and non-ischemic cardiomyopathy in NYHA class III-IV. This is documented for stable patients on optimal medical therapy with dilated LV, LVEF ≤ 35% and QRS width > 120 ms [5]. The benefit of CRT in cardiogenic shock has not been studied. Some observational studies have reported beneficial outcome from CRT in inotrope-supported patients with end-stage heart failure [7,8], and there are case reports on clinical improvement effected by CRT in patients on IABP support [9]. The rapid onset of hemodynamic improvement of CRT may be of clinical benefit in an acute setting and it is likely that CRT has an additive effect on the unloading of the left ventricle and improved organ perfusion achieved by the LVAD in patient with cardiogenic shock. This should be judged against the elevated risk of complications using mechanical devices in this group of unstable patients. The use of LVAD and CRT combined in cardiogenic shock has to our knowledge not been reported previously.

## Consent

Written informed consent was obtained from the patient for publication of this case report and any accompanying images.

## Competing interests

The authors declare that they have no competing interests.

## Authors' contributions

All authors critically read, discussed and approved the final draft of the manuscript.
